# Anxiety, depression and quality of life in acute high risk cardiac disease patients eligible for wearable cardioverter defibrillator: Results from the prospective multicenter CRED-registry

**DOI:** 10.1371/journal.pone.0213261

**Published:** 2019-03-11

**Authors:** Michael Weiss, Guido Michels, Frank Eberhardt, Wolfgang Fehske, Stefan Winter, Frank Baer, Yeong-Hoon Choi, Christian Albus, Daniel Steven, Stephan Baldus, Roman Pfister

**Affiliations:** 1 Department III of Internal Medicine, Heart Center, University of Cologne, Cologne, Germany; 2 Evangelisches Krankenhaus Köln Kalk, Cologne, Germany; 3 St. Vinzenz Hospital, Cologne, Germany; 4 St. Antonius Krankenhaus, Cologne, Germany; 5 Klinik für Herz- und Thoraxchirurgie, Heart Center, University of Cologne, Cologne, Germany; 6 Department of Psychosomatics and Psychotherapy, University of Cologne, Cologne, Germany; University of Messina, ITALY

## Abstract

**Background:**

Psychological distress is common in patients with cardiovascular disease and negatively impacts outcome.

**Hypothesis:**

Psychological distress is high in acute high risk cardiac patients eligible for a WCD, and associated with low quality of life. Distress is aggravated by WCD.

**Methods:**

Consecutive patients eligible for a WCD were included in the prospective, multicenter “Cologne Registry of External Defibrillator” registry. Quality of life (Short Form-12), depressive symptoms (Beck-Depression Inventory II) and anxiety (State Trait Anxiety Inventory) were assessed at enrollment and 6-weeks, and associations with WCD prescription were analyzed.

**Results:**

123 patients (mean [SD] age 59 [± 14] years, 75% male) were included, 85 (69%) of whom received a WCD. At enrollment 21% showed clinically significant depressive symptoms and 52% anxiety symptoms, respectively. At 6 weeks, depressive and anxious symptoms significantly decreased to 7% and 25%, respectively. Depressive symptoms at enrollment and changes at 6 weeks showed significant associations with health-related quality of life, whereas anxious symptoms did not. There was a trend for better improvement of depression scores in patients with WCD (mean [SD] change in score points: -4.1 [6.1] vs -1.8 [3.9]; p = 0.09), whereas change of the anxiousness score was not different (-4.6 [9.5]) vs -3.7 [9.1], p = 0.68).

**Conclusion:**

In patients eligible for a WCD, depressive and anxiety symptoms were initially common and depressive symptoms showed a strong association with reduced health-related quality of life contributing to their clinical relevance. WCD recipients showed at least similar improvement of depression and anxiety at 6 weeks when compared to non recipients.

## Introduction

Psychological distress is associated with the development and prognosis of cardiovascular disease, and receives increasing attention in clinical care [1, 2]. About one in five patients with chronic heart failure has symptoms of depression, and presence of depression is associated with increased risk of hospitalization and mortality [3, 4]. Similar findings were reported for anxiety, with a strong mutual correlation between depression and anxiety [5, 6]. Notably, both factors substantially contribute to the severely impaired health related quality of life in patients with chronic heart failure [7]. Patients who are at risk of or who experienced ventricular tachy-arrhythmias are also relevantly affected by psychological distress. In patients with implantable cardioverter-defibrillator (ICD)-implantation 9–18% and 16–36% showed depression and anxiety, respectively [8, 9].

The detection of psychological distress in patients with heart failure and arrhythmias is of clinical interest for several reasons. First, it is a strong potentially modifiable determinant of impaired quality of life. Second, psychological distress may impact outcome. Depression is a predictor of mortality and appropriate shocks in patients with ICD [10, 11], and there is ongoing discussion on whether depression and anxiety might causally increase arrhythmias through effects on autonomic nervous system [12]. Third, psychological distress might also affect medication adherence and life-style, both of which strongly determine outcome in cardiovascular patients [13].

So far, data within the acute setting of first diagnosis of left-ventricular dysfunction and increased arrhythmic risk are lacking. Due to the sudden onset of such severe disease we hypothesized that those patients might have high rate of psychological distress and this might also affect patient`s quality of life. Given the strong potential impact of psychological distress on the clinical course of cardiovascular patients, the aim of this study was to assess the prevalence of depressive and anxiety symptoms and the association with quality of life in the setting of acute cardiovascular disease and increased risk of ventricular arrhythmias. Furthermore, we sought to examine whether the psychological distress is confined to the acute disease manifestation or persists during short-term follow-up. Finally, we aimed to assess whether the temporal change of psychological distress differs with the prescription of a wearable cardioverter defibrillator (WCD). The latter is prescribed in clinical routine for selected patients for surveillance and potential treatment of arrhythmias. Psychological distress in WCD carriers has not been reported so far. However, an impact on psychological distress could be expected from WCD in both directions, through an increase of disease and risk awareness resulting from daily wearing, or an increased feeling of safety provided by the life-saving treatment capability.

## Methods

### Patients

The prospective, multicenter “Cologne registry of external defibrillation” (CRED) was initiated in 01/05/2014 in five cardiological hospital departments in the city of Cologne. Consecutive patients were screened for presence of increased risk of ventricular arrhythmias and sudden cardiac death as potential candidates for a WCD according to pre-specified criteria adapted from the Heart and Rhythm Society. Briefly, patients had either (1) an ICD explanted due to e.g. infection and were waiting for re-implantation, or had an indication for ICD but implantation had to be postponed due to ventricular thrombus, or (2) first diagnosis of heart failure with reduced ejection fraction (EF<35%) just starting disease modifying therapy, or first diagnosis of left-ventricular dysfunction (EF<35%) due to disease with high likelihood of recovery such as myocarditis, peripartum cardiomyopathy or takotsubo cardiomyopathy, or (3) ischemic cardiomyopathy with EF<35% in the setting of acute myocardial infarction and/or revascularization with percutaneous coronary intervention or coronary artery bypass. Exclusion criteria were age below 18 years, missing consent and severe language or intellectual deficits precluding an interview on medical history and psychological distress measures. Since clear criteria for the use of WCD were lacking at the time of study initiation, the decision to use a WCD or not was finally made by the treating physician based on his subjective risk-benefit judgement prior to discharge of the patient. The main reason given by the physician for not prescribing the WCD was assessed in the registry. The patients were not informed about the WCD option in case prescription was not recommended by the physician. Aim of CRED was to prospectively compare baseline characteristics and clinical outcome of patients with a WCD to those without a WCD in order to provide insight on decision criteria of physicians. The study was approved by the local ethics committee of the University hospital of Cologne (14–050; trial registration NCT02073942) and was conducted according to the principles expressed in the Declaration of Helsinki. All patients provided written informed consent.

### Data collection and follow-up

At baseline demographic and clinical data were extracted from the patient record. Patients underwent a baseline face-to-face interview on health-related quality of life (Short Form-12 Version 2 in German Language, OptumInsight LifeSciences) and depressive symptoms (Beck-Depression Inventory [BDI]-version II in German Language, applied to the patient`s symptoms over the previous 2 weeks, Person Assessment & Information GmbH), and a self-report questionnaire on anxiety (State Trait Anxiety Inventory in German Language, Beltz Test GmbH), all according to the recommendations of the provider. The interview was performed by a trained medical student, who was blinded to clinical data. This psychological assessment was performed before the option of WCD was discussed with the patient.

Responses to the SF-12 applied to the patient’s health over the previous 4 weeks and were graded and scored from 0 to 100, with a higher score reflecting better health-related quality of life. Two separate component summary scores are provided, distinguishing between physical (physical component score [PCS]) and mental (mental component score [MCS]) health. The State Trait Anxiety Inventory (STAI) has well established validity and reliability [14] and assesses both state and trait anxiety, where trait anxiety reflects how a person generally feels and state anxiety reflects how the patient was feeling at the time of the assessment. The latter was used for primary analyses and was analysed both as a continuous score to detect minor changes in anxiety symptoms, and with aa cut-off of >40 to assess the prevalence of relevant anxiety symptoms [15]. BDI-II also has well established validity and reliability [16]. Depression severity was categorized according to the BDI-II manual: absent (0–13), mild (14–19), moderate (20–28), or severe (≤29).

A clinical follow-up with repeated questionnaire assessment was performed at 6 weeks, again before the patient was informed on the development of cardiac function. The 6 week interval for psychological reassessment was chosen so that patients could get used to the WCD but still were before the decision on definitive treatments such as ICD which usually occurs at three months. Thus, the psychological assessment should reflect the steady state on or off WCD.

### Statistical analysis

Continuous variables were expressed as means (standard deviation) or median (interquartile range) and groups by WCD status were compared using T-test or Wilcoxon-Mann-Whitney-test as appropriate. Categorical data were summarized as frequencies and percentages and groups by WCD status were compared using chi2 test. Temporal changes of the score measures between baseline and 6-week follow-up were compared using paired T-test and chi2 test. The association between psychological measures and quality of life was analysed using linear regression analysis with the former as dependent variable. Multivariable regression analysis was performed including all baseline characteristics with a significant association (p<0.05) with psychological measures using stepwise backward elimination.

The association of WCD with baseline score values and prevalences of depression and anxiety was examined by linear and logistic regression analysis with the distress measures as dependent variables, adjusting for baseline characteristics which differed significantly between patients with and without WCD. The regression analysis of WCD on absolute changes of distress measures was additionally adjusted for the baseline values of the respective scores. Patients with a WCD prescription were analysed in the WCD group as intention-to-treat, no matter whether WCD was terminated before follow-up or not. To identify a potential impact on distress associated with early WCD termination, 6 week depression and anxiety scores were compared between patients with early WCD termination and persistent carriers.

All statistical tests were two-sided, and a nominal p-value of p<0.05 was considered statistically significant. Analyses were carried out with SPSS software (version 24).

## Results

**Fig 1:**
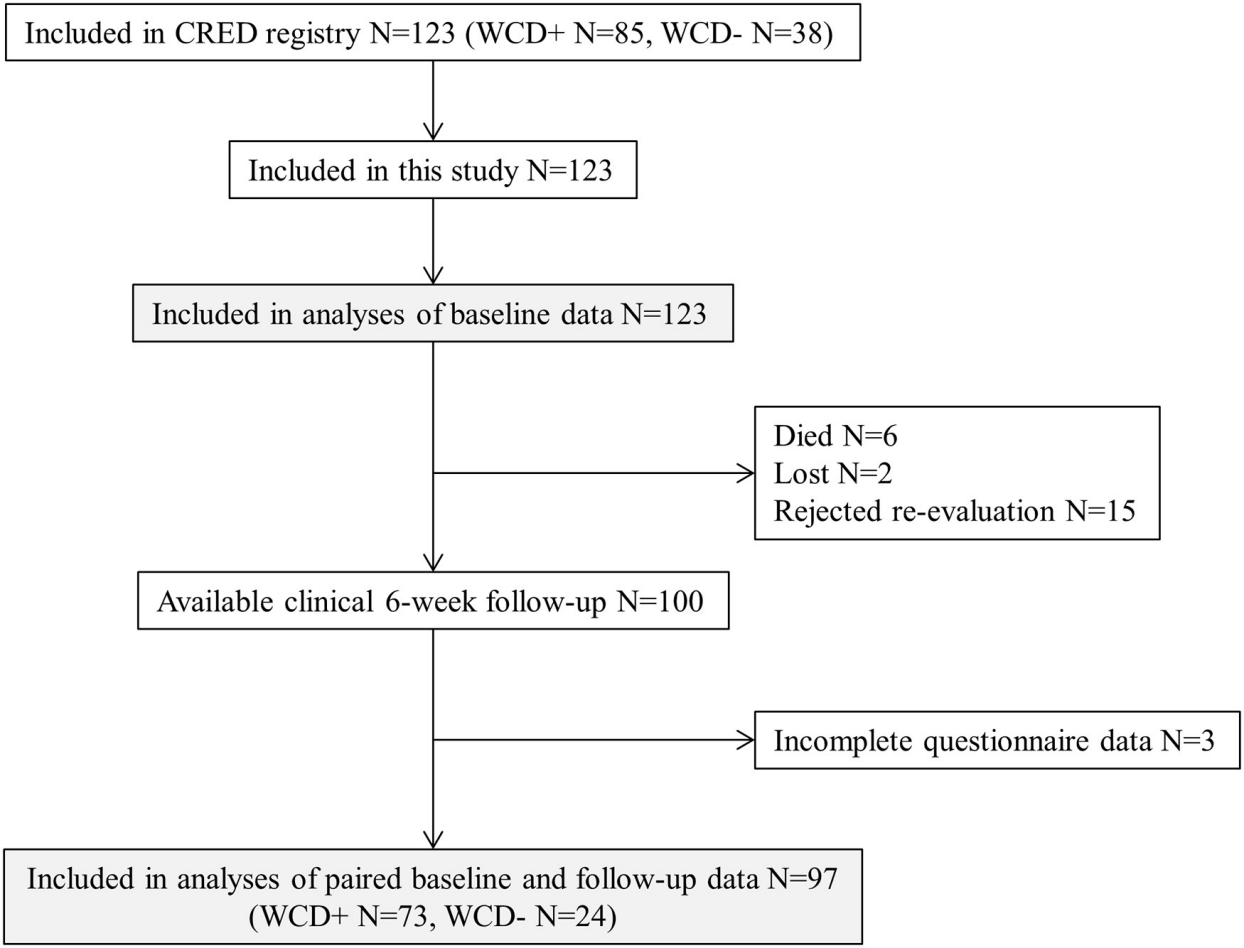
Flow chart of the study population.

123 patients were included in the registry and in this analysis. Baseline characteristics are shown in Table 1. Mean (SD) age of the patients was 59 (± 14) years and 75% were male. Six weeks’ clinical follow-up examination with complete questionnaire data was available in 97 (79%) patients, with 2 patients lost to follow-up, 6 patients deceased within first 6 weeks, 15 patients rejecting follow up examination and 3 patients with incomplete follow-up questionnaire data (Fig 1). Patients with complete paired questionnaire data (n = 97) did not differ relevantly regarding baseline characteristics compared to those alive without complete questionnaire data (n = 20) (S1 Table). In particular, there was no statistically significant difference in health-related quality of life, depression measures and anxiety rate.

**Table 1:** Baseline characteristics of the total population and by status of WCD prescription (n = 123)^a^.

	WCD therapy	No WCD therapy	p-value	Total population
**n**	85	38		123
**Age [years ± SD]**	56 ± 13	64 ± 14	0.01	59 ± 14
**Female gender [%]**	20 (n = 18)	34 (n = 13)	0.18	25 (n = 31)
**Married [%]**	14 (n = 12)	17 (n = 6)	0.78	15 (n = 18)
**At least certificate of secondary education [%]**	48 (n = 41)	26 (n = 10)	0.03	42 (n = 51)
**Prior history of depression [%]**	6 (n = 5)	13 (n = 5)	0.28	8 (n = 10)
**Current intake of psychotropic medication**^b^ **[%]**	14 (n = 12)	18 (n = 7)	0.59	15 (n = 19)
**Indication**				
**Temporarily explanted ICD/ postponed ICD implantation [%]**	9 (n = 8)	8 (n = 3)	1.00	9 (n = 11)
**Ischemic heart disease [%]**	24 (n = 20)	39 (n = 15)	0.09	29 (n = 35)
**With acute myocardial infarction [%]**	22 (n = 19)	39 (n = 15)	0.08	28 (n = 34)
**Non ischemic cardiomyopathy [%]**	67 (n = 57)	52 (n = 20)	0.16	63 (n = 77)
**Comorbidity**				
**Hypertension [%]**	54 (n = 46)	47 (n = 18)	0.56	52 (n = 64)
**Hyperlipoproteinaemia [%]**	14 (n = 12)	21 (n = 8)	0.43	16 (n = 20)
**Prior stroke [%]**	4 (n = 3)	8 (n = 3)	0.37	5 (n = 6)
**Diabetes mellitus [%]**	26 (n = 23)	21 (n = 8)	0.65	25 (n = 31)
**Prior myocardial infarction [%]**	22 (n = 19)	40 (n = 15)	0.08	27 (n = 34)
**Current smoker [%]**	65 (n = 55)	58 (n = 22)	0.47	63 (n = 77)
**Any regular alcohol intake [%]**	31 (n = 27)	31 (n = 12)	1.00	31 (n = 39)
**Prior or active malignancy [%]**	2 (n = 2)	13 (n = 5)	0.03	6 (n = 7)
**Arrhythmic risk profile** **(syncope or family history of sudden cardiac death or prior resuscitation/ventricular tachycardia/ventricular fibrillation) [%]**	31 (n = 26)	26 (n = 10)	0.63	29 (n = 36)
**Previous arrhythmia (resuscitation, ventricular tachycardia or fibrillation) [%]**	18 (n = 15)	11 (n = 4)	0.42	15 (n = 19)
**Clinical data**				
**NYHA class [± SD]**	3.0 ± 0.8	3.1 ± 0.6	0.42	3.0 ± 0.8
**Left-ventricular ejection fraction [% ± SD]**	26 ± 8	25 ± 7	0.73	26 ± 8
**Heart rate [1/min ± SD]**	80 ± 18	82 ± 14	0.57	81 ± 17
**Systolic blood pressure [mmHg ± SD]**	116 ± 18	119 ± 21	0.40	117 ± 19
**Dystolic blood pressure [mmHg ± SD]**	73 ± 14	73 ± 11	0.94	73 ± 13
**Body mass index [kg/m**^**2**^ **± SD]**	28 ± 6	26 ± 5	0.25	27 ± 6
**Trait anxiety [± SD]**	36.7 ± 9.9	35.1 ± 10.5	0.42	36.2 ± 10.1
**Laboratory parameters**				
**Glomerular filtration rate** **[ml/min/1.73m**^**2**^ **± SD**	72 ± 26	62 ± 28	0.09	69 ± 27
**NT-pro-BNP [ng/l ± SD]**	6206 ± 9041	11317 ± 12307	0.08	7969 ± 10467
**TSH [± SD]**	2.30 ± 2.13	4.39 ± 11.73	0.35	2.88 ± 6.44
**Drug treatment**				
**ACE-inhibitor or angiotensin-receptor blocker [%]**	93 (n = 79)	90 (n = 34)	0.50	92 (n = 114)
**Beta-blocker [%]**	88 (n = 75)	74 (n = 29)	0.06	84 (n = 104)
**Mineralcorticoidreceptor antagonist [%]**	69 (n = 59)	63 (n = 24)	0.54	68 (n = 83)
**Thiazide or loop diuretics [%]**	79 (n = 67)	76 (n = 28)	0.81	78 (n = 95)

ACE: angiotensin converting enzyme, ICD: implantable cardioverter defibrillator, NT-pro-BNP: N-terminal-pro-brain natriuretic peptide, NYHA: New York Heart Association, TSH: thyroid stimulating hormone, WCD: wearable cardioverter defibrillator

^a^ data are presented as mean ± standard deviation (SD) or percentage [frequency n]

^b^ selective serotonin reuptake inhibitors, benzodiazepines, low and high potency antipsychotics, tricyclic and other antidepressants

**Fig 2:**
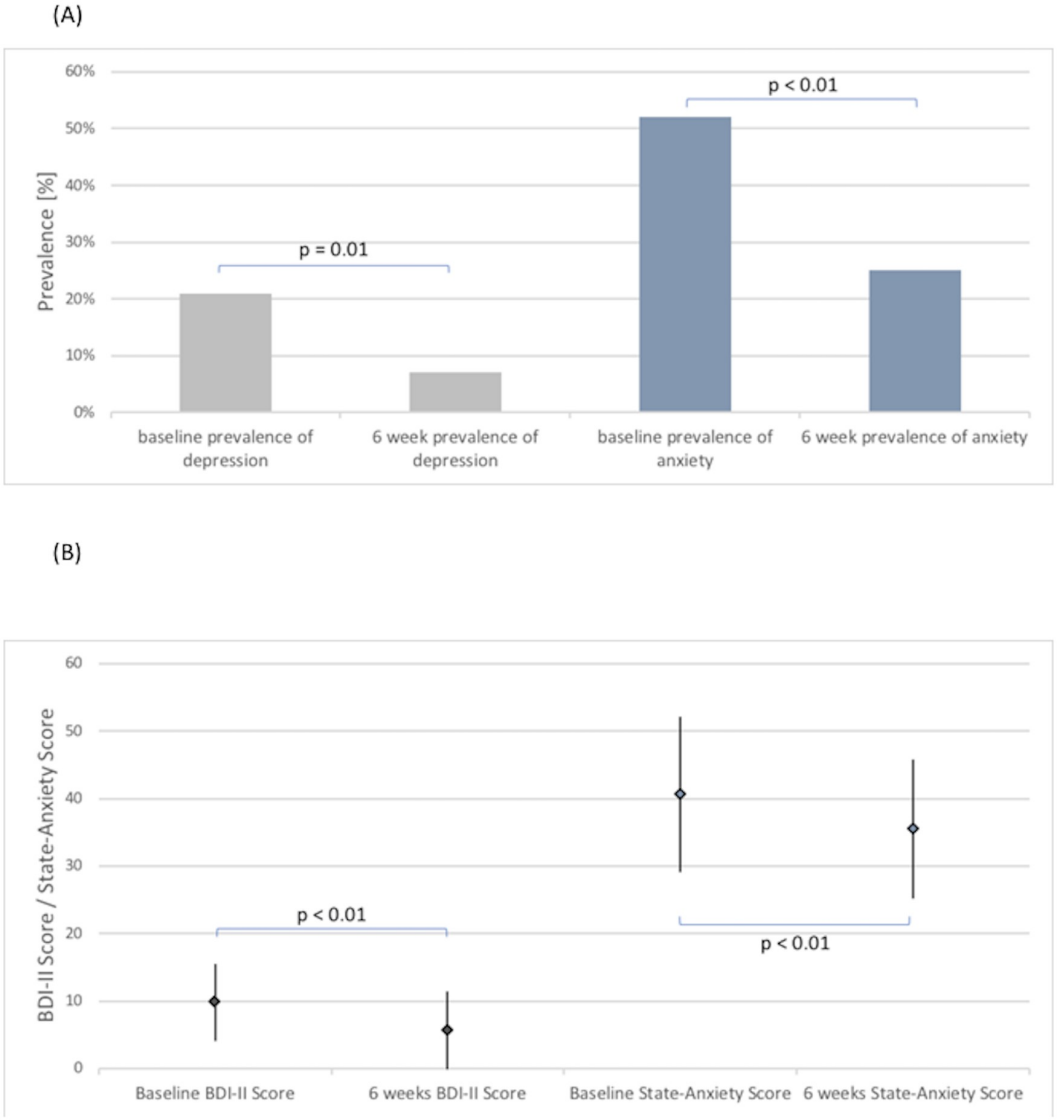
Baseline and 6-week prevalences (A) and score values (B) of depression and anxiety (n = 97).

At baseline 21% of patients showed at least mild depressive symptoms and 52% of patients showed symptoms of anxiety (Fig 2), with 17% of all patients showing concomitant depressive and anxiety symptoms.

Mean BDI score at baseline was 10 (± 5). There was a significant inverse association with physical and mental component scores (both p<0.01 for linear regression analysis), which was slightly attenuated but still significant when adjusting for other significant baseline predictors of the BDI score such as NYHA class, prior history of mental disease, current intake of psychotropic medication, alcohol intake, and state and trait anxiety (p<0.01 and p = 0.03, respectively)(S1 Fig). At 6 weeks, the mean BDI score significantly decreased to 6 (±6, p<0.001) and the frequency of depressive symptoms also significantly decreased from 21% to 7% (p = 0.004, Fig 2A). The change in BDI score was also inversely and significantly associated with changes in mental and physical component score (S2 Fig, p-values <0.01 for adjusted linear regression analysis).

Mean state anxiety score was 41 (± 12). The association with physical and mental component scores was of borderline significance (p = 0.09 and p = 0.13 for linear regression analysis). At 6 weeks, the mean anxiety score significantly decreased to 36 (± 10, p<0.001) and the rate of clinically significant anxiousness significantly decreased from 52% to 25% (p<0.001, Fig 2B). The change in anxiety score was not associated with changes of physical and mental component scores.

The frequencies and temporal changes were virtually the same when excluding patients with acute myocardial infarction, ICD explantation and indication for ICD but postponed implantation (S3 Fig).

### Associations with WCD

A WCD was finally prescribed in 85 (69%) patients after the baseline assessment was completed. Reasons for not prescribing a WCD were motoric or intellectual deficits precluding appropriate use of the WCD in 5 patients, a low risk of sudden cardiac death estimated by the treating physicians in 31 patients, and unspecified in 2 patients. Patients who were prescribed a WCD did not differ significantly from those without WCD except for younger age, higher educational level and lower rate of malignancy (Table 1). The prescription, dosage and change of dosage from baseline of ACE-inhibitors/angiotensin receptor blockers and beta-blockers and the improvement of left-ventricular ejection fraction did not differ significantly between patients with and without WCD. The WCD was prescribed for a median of 59 days (IQR 40–96 days), and the mean daily wearing time was 20 (±5) hours. 22 patients terminated WCD wearing before follow-up assessment at 6 weeks, 11 patients because of improved LV function assessed by their cardiologist, 9 patients because of early ICD implantation (5 of whom were patients with previous ICD explantation or postponed implantation) and 2 patients for own will. Patients with WCD did not have any treatment for tachyarrhythmia or inappropriate shocks by the WCD.

**Fig 3:**
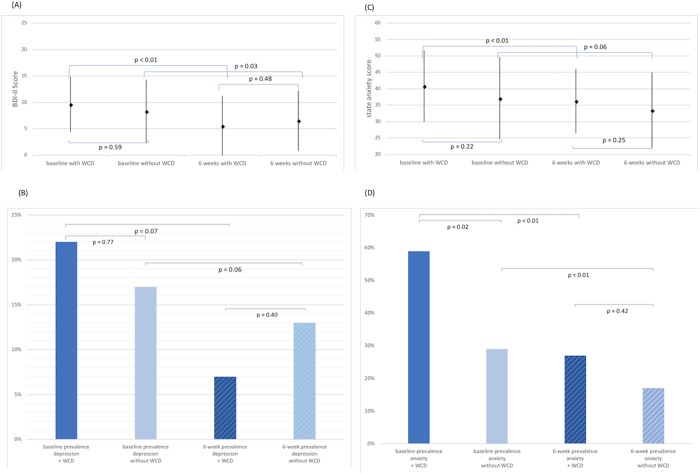
Baseline and 6-weeks depressive (A+B) and anxiety (C+D) symptoms by WCD use (n = 97).

Prevalence of clinically significant depression and anxiety and mean BDI and anxiety scores at baseline and 6 weeks by WCD are shown in Fig 3. Patients with subsequent WCD prescription showed a higher baseline state anxiety score (41 ± 11) compared to those without WCD (39 ± 13, p = 0.22), and had a significantly higher rate of anxiety (58.9% versus 29.2%, p = 0.02). The association between WCD and baseline anxiety was still significant when adjusting for significant differences in baseline characteristics between patients with and without WCD such as age, education level and history of malignancy (p = 0.02). Overall, temporal changes of depression and anxiety were similar in patients with and without WCD, with a trend for a more pronounced improvement of depression in the WCD group. The mean change of BDI score was -4.1 (±6.1) and -1.8 (±3.9) in patients with and without WCD. The mean changes of the anxiety score were -4.6 (±9.5) and -3.7 (±9.1) in patients with and without WCD. In a linear regression analysis adjusted for baseline values of the respective score, age, education level and history of malignancy, there was a trend towards greater reduction of depressive symptoms in the WCD group (p = 0.09), whereas no association with anxious symptoms could be seen (p = 0.68). The frequencies and temporal changes were virtually the same when excluding patients with acute myocardial infarction, ICD explantation and indication for ICD but postponed implantation (S4 Fig). Mean daily wearing time of WCD was not significantly associated with baseline BDI or state anxiety score and changes of both scores from baseline to 6 weeks in linear regression analysis. BDI and state anxiety score at 6 weeks did not differ significantly between patients with persistent wearing of the WCD and those who terminated WCD wearing before the assessment (both p>0.05).

## Discussion

In this prospective multicenter cohort of patients with acute cardiovascular disease and increased risk of sudden cardiac death eligible for WCD we found a high prevalence of psychological distress in the acute setting. Depression and anxiety were highly correlated, and depression also showed strong association with health-related quality of life. Six weeks after the index event, depressive and anxiety symptoms substantially improved, which was accompanied by improvement in quality of life. Patients subsequently prescribed a WCD had a higher prevalence of anxiety at baseline compared to patients without WCD, which was not attributable to differences in available clinical baseline characteristics. There is weak evidence that WCD is associated with reduced depressiveness during 6 weeks of follow-up compared to no WCD, whereas there was no association for anxiety.

Our acutely diseased cardiovascular patients had higher scores indicating more depressive and anxious symptoms than healthy reference populations, for which mean BDI values of 7 to 8 and mean anxiety score of 34 to 37 are reported [17, 18]. This extends the body of literature on increased psychological distress in patients with chronic cardiovascular disease for a well defined population of acute cardiovascular patients. Absolute prevalence data of psychological distress for cardiovascular populations show substantial heterogeneity, which to a large part is attributable to differing assessment tools [3]. Patients with stable chronic heart failure, where identical assessment tools were used as in our study, showed both lower BDI and anxiety score [19, 20]. Patients with chronic heart failure participating in the Sudden Cardiac Death in Heart Failure trial (SCD-HEFT) showed a mean BDI score of 11 and an anxiety score of 37, which is comparable or lower than in our population [6]. In patients undergoing first ICD implantation the rate of depressive symptoms was 23%, similar to our cohort, and rate of anxiety was 37%, which is still lower than in our cohort [21]. Taken together, the combination of severe cardiac disease with high symptom burden and high arrhythmic risk, manifested acutely to the majority of our patients, might explain the higher rate of psychological distress in our cohort compared to more chronically diseased patients. The prevalence of depression [22, 23] and anxiety [24] in patients manifesting with acute myocardial infarction was of similar magnitude as in our cohort. Notably, prevalence of depression and anxiety were virtually identical in our study when excluding patients with acute myocardial infarction from the analysis, underlining the substantial psychological stress patients are exposed to after first diagnosis of severe left-ventricular dysfunction independently of myocardial ischemia. The strong and independent association between depression and measures of physical and mental functioning which is well known from patients with chronic stable cardiovascular disease was also observed in our acutely manifested patients [1].

Data on temporal changes of psychological distress following acute cardiac events are sparse. In patients undergoing elective coronary intervention, significant improvements were observed only three to six months post procedure [25]. In contrast, patients with acute myocardial infarction undergoing coronary intervention showed a significant improvement of depressive and anxiety already several days after the procedure [22]. Our patients might be in a more complex clinical situation. During initial assessment, our patients were physically compromised by heart failure or acute ischemic heart disease symptoms, and mentally by first diagnosis of severe cardiac disease and arrhythmic risk. The substantial early improvement of psychological distress might be explained by a symptom relief after initiating treatment and improvement of LV function. Patients with acute heart failure show substantial improvement of symptoms within the first 30 days [26]. In line with this, the strong improvement in physical capacity assessed by SF12 correlated well with depression improvement. Whether additional coping processes might also contribute to improved psychological distress can only be speculated. The improvement of anxiety was not associated with improvement of physical capacity suggesting additional underlying mechanisms.

To the best of our knowledge, associations of WCD with psychological measures have not been examined so far. We observed a substantial decrease of anxiety and depression after the first 6 weeks in WCD patients which was similar as in the subgroup of patients without WCD. Given the non-randomized design of our study, it is impossible to estimate the causal contribution of WCD to the changes of psychological distress, and the comparability of both groups is limited as shown by the higher baseline prevalence of anxiety in the WCD group. However, after adjusting the analysis to address these differences in baseline characteristics as well as the difference in baseline score values between patients with and without WCD, there was an association of borderline significance between WCD and changes of depression, but not with anxiety. Thus, WCD is clearly not associated with increased anxiety and depression, but may have also positive impact on depressive symptoms. This does not contribute to the hypothesis that, in analogy to patients with ICD treatment, WCD might remember the patient of his life-threatening cardiac disease and the anticipation of shocks, thus triggering phobic anxiety and depressed mood [27]. In contrast to ICD treatment [28, 29], due to the exposed wearing compared to ICDs, the WCD might enable patients to feel more secure.

Several limitations of this study should be considered. The sample size did not allow analysis of associations with clinical outcome. Important for the interpretation of our findings is the fact that none of our patients with WCD experienced a shock therapy. This is statistically not unexpected given a therapy rate of WCD in large registries of less the 2% [30]. Considering results from ICD patients, shock therapy might have strong impact on psychological distress [31]. WCD allocation was not randomized, which limits a conclusion on causality of effects observed in the WCD group. Important to note though, this is the first study which provides a prospectively selected control group for WCD patients at all. Furthermore, we did not assess new initiation of psychotropic medication or psychological therapy at 6 weeks, which might have contributed to the improvement of depressive and anxiety symptoms. The results of the psychological questionnaire assessments were blinded to treating physicians with the exception of one patient who showed severe depressive symptoms. This patient underwent initial psychiatric assessment as a consequence of our study assessment. Since this patient finally rejected follow-up assessment at 6 weeks, data were not considered for analysis of temporal changes. Hence, the likelihood of interim psychological interventions contributing to the improvement of distress is low, given the short follow-up time where logistically it will be difficult to initiate psychological therapy. Finally, the BDI score might not be the optimal measure for assessing longitudinal changes of depressive symptoms in patients without history of major depressions. The choice to use BDI was made pragmatically since several studies with heart failure or ICD patients also used BDI in the past and hence comparison of results across studies was possible.

## Conclusion

Depressive and anxiety symptoms are common in acute cardiovascular patients at increased risk of sudden cardiac death, with prevalences comparable to heart failure patients with ICD implantation or acute myocardial infarction, but improve markedly during 6 weeks follow-up. The depressive symptoms during the acute disease manifestation as well as the short-term improvement are strongly associated with patients`health-related quality of life indicating the clinical relevance for the patient. Acknowledging the non-randomized design of this study, we found preliminary evidence that WCD may alleviate depressive symptoms more than treatment without WCD. Further large scale prospective trials with longer follow-up are needed to reevaluate these findings.

## Supporting information

S1 Fig Correlation of baseline BDI-II score with baseline MCS (A) and baseline BDI-II score with baseline PCS (B) (n = 123). (DOCX)

S2 Fig Correlation of ΔMCS with ΔBDI-II score (A) and ΔPCS and ΔBDI-II score (B) (n = 97). (DOCX)

S3 Fig Baseline and 6-week depression (A) and anxiety (B) in patients without acute myocardial infarction, ICD explantation and ICD indication with postponed implantation (N = 71). (DOCX)

S4 Fig Baseline and 6-weeks depressive (A+B) and anxiety (C+D) symptoms by WCD therapy in patients without acute myocardial infarction, ICD explantation and ICD indication with postponed implantation (N = 71). (DOCX)

S1 Table Baseline characteristics in patients with available baseline and follow-up questionnaire data and those alive without complete questionnaire data. (DOCX)

## References

[1] Ladwig KH, Lederbogen F, Albus C, Angermann C, Borggrefe M, Fischer D, et al Position paper on the importance of psychosocial factors in cardiology: Update 2013. Ger Med Sci. 2014;12:Doc09 Epub 2014/05/09. 10.3205/000194 . 24808816 PMC4012565

[2] Piepoli MF, Hoes AW, Agewall S, Albus C, Brotons C, Catapano AL, et al 2016 European Guidelines on cardiovascular disease prevention in clinical practice: The Sixth Joint Task Force of the European Society of Cardiology and Other Societies on Cardiovascular Disease Prevention in Clinical Practice (constituted by representatives of 10 societies and by invited experts)Developed with the special contribution of the European Association for Cardiovascular Prevention & Rehabilitation (EACPR). Eur Heart J. 2016;37(29):2315–81. 10.1093/eurheartj/ehw106 . 27222591 PMC4986030

[3] Rutledge T, Reis VA, Linke SE, Greenberg BH, Mills PJ. Depression in heart failure a meta-analytic review of prevalence, intervention effects, and associations with clinical outcomes. J Am Coll Cardiol. 2006;48(8):1527–37. Epub 2006/10/19. 10.1016/j.jacc.2006.06.055 . 17045884

[4] Adelborg K, Schmidt M, Sundboll J, Pedersen L, Videbech P, Botker HE, et al Mortality Risk Among Heart Failure Patients With Depression: A Nationwide Population-Based Cohort Study. J Am Heart Assoc. 2016;5(9). 10.1161/JAHA.116.004137 . 27604456 PMC5079053

[5] Dekker RL, Lennie TA, Doering LV, Chung ML, Wu JR, Moser DK. Coexisting anxiety and depressive symptoms in patients with heart failure. Eur J Cardiovasc Nurs. 2014;13(2):168–76. 10.1177/1474515113519520 . 24408885 PMC3992982

[6] Friedmann E, Thomas SA, Liu F, Morton PG, Chapa D, Gottlieb SS, et al Relationship of depression, anxiety, and social isolation to chronic heart failure outpatient mortality. Am Heart J. 2006;152(5):940 e1–8. Epub 2006/10/31. 10.1016/j.ahj.2006.05.009 . 17070164

[7] Uchmanowicz I, Gobbens RJ. The relationship between frailty, anxiety and depression, and health-related quality of life in elderly patients with heart failure. Clin Interv Aging. 2015;10:1595–600. 10.2147/CIA.S90077 . 26491276 PMC4599570

[8] Thylen I, Dekker RL, Jaarsma T, Stromberg A, Moser DK. Characteristics associated with anxiety, depressive symptoms, and quality-of-life in a large cohort of implantable cardioverter defibrillator recipients. J Psychosom Res. 2014;77(2):122–7. 10.1016/j.jpsychores.2014.05.007 . 25077853

[9] Gostoli S, Bonomo M, Roncuzzi R, Biffi M, Boriani G, Rafanelli C. Psychological correlates, allostatic overload and clinical course in patients with implantable cardioverter defibrillator (ICD). Int J Cardiol. 2016;220:360–4. 10.1016/j.ijcard.2016.06.246 . 27390955

[10] Whang W, Albert CM, Sears SF Jr., Lampert R, Conti JB, Wang PJ, et al Depression as a predictor for appropriate shocks among patients with implantable cardioverter-defibrillators: results from the Triggers of Ventricular Arrhythmias (TOVA) study. J Am Coll Cardiol. 2005;45(7):1090–5. Epub 2005/04/06. 10.1016/j.jacc.2004.12.053 . 15808769

[11] Mastenbroek MH, Versteeg H, Jordaens L, Theuns DA, Pedersen SS. Ventricular tachyarrhythmias and mortality in patients with an implantable cardioverter defibrillator: impact of depression in the MIDAS cohort. Psychosom Med. 2014;76(1):58–65. 10.1097/PSY.0000000000000017 . 24336430

[12] Francis JL, Weinstein AA, Krantz DS, Haigney MC, Stein PK, Stone PH, et al Association between symptoms of depression and anxiety with heart rate variability in patients with implantable cardioverter defibrillators. Psychosom Med. 2009;71(8):821–7. 10.1097/PSY.0b013e3181b39aa1 . 19661191 PMC2794038

[13] Gehi A, Haas D, Pipkin S, Whooley MA. Depression and medication adherence in outpatients with coronary heart disease: findings from the Heart and Soul Study. Arch Intern Med. 2005;165(21):2508–13. 10.1001/archinte.165.21.2508 . 16314548 PMC2776695

[14] Spielberger C D, G RL, Lushene R. E. The State-Trait Anxiety Inventory (test manual). Palo Alto, CA: Consulting Psychologists Press; 1970.

[15] Frasure-Smith N, Lesperance F, Talajic M. The impact of negative emotions on prognosis following myocardial infarction: is it more than depression? Health Psychol. 1995;14(5):388–98. Epub 1995/09/01. . 7498109 10.1037//0278-6133.14.5.388

[16] Beck AT SR, Brown GK. The Beck Depression Inventory-second edition manual. San Antonio: The Psychological Corporation; 1996.

[17] Laux L GP, Schaffer P, Spielberger CD. Das State-Trait Angstinventar Manual.

[18] Hautzinger M KF, Kühner C. BDI-II Beck Depressions-Inventar Revision Manual.

[19] Blumenthal JA, Babyak MA, O'Connor C, Keteyian S, Landzberg J, Howlett J, et al Effects of exercise training on depressive symptoms in patients with chronic heart failure: the HF-ACTION randomized trial. JAMA. 2012;308(5):465–74. Epub 2012/08/02. 10.1001/jama.2012.8720 . 22851113 PMC3953459

[20] Jiang W, Kuchibhatla M, Cuffe MS, Christopher EJ, Alexander JD, Clary GL, et al Prognostic value of anxiety and depression in patients with chronic heart failure. Circulation. 2004;110(22):3452–6. Epub 2004/11/24. 10.1161/01.CIR.0000148138.25157.F9 . 15557372

[21] Dunbar SB, Langberg JJ, Reilly CM, Viswanathan B, McCarty F, Culler SD, et al Effect of a psychoeducational intervention on depression, anxiety, and health resource use in implantable cardioverter defibrillator patients. Pacing Clin Electrophysiol. 2009;32(10):1259–71. Epub 2009/10/03. 10.1111/j.1540-8159.2009.02495.x . 19796343 PMC2757745

[22] Kala P, Hudakova N, Jurajda M, Kasparek T, Ustohal L, Parenica J, et al Depression and Anxiety after Acute Myocardial Infarction Treated by Primary PCI. PLoS One. 2016;11(4):e0152367 Epub 2016/04/14. 10.1371/journal.pone.0152367 . 27074002 PMC4830576

[23] Thombs BD, Notes LD, Lawrence JW, Magyar-Russell G, Bresnick MG, Fauerbach JA. From survival to socialization: a longitudinal study of body image in survivors of severe burn injury. J Psychosom Res. 2008;64(2):205–12. Epub 2008/01/29. 10.1016/j.jpsychores.2007.09.003 . 18222134

[24] Haberka M, Mizia-Stec K, Mizia M, Gieszczyk K, Chmiel A, Sitnik-Warchulska K, et al Effects of n-3 polyunsaturated fatty acids on depressive symptoms, anxiety and emotional state in patients with acute myocardial infarction. Pharmacol Rep. 2013;65(1):59–68. Epub 2013/04/09. . 23563024 10.1016/s1734-1140(13)70964-2

[25] Gu G, Zhou Y, Zhang Y, Cui W. Increased prevalence of anxiety and depression symptoms in patients with coronary artery disease before and after percutaneous coronary intervention treatment. BMC Psychiatry. 2016;16:259 10.1186/s12888-016-0972-9 . 27450548 PMC4957885

[26] Sauser K, Spertus JA, Pierchala L, Davis E, Pang PS. Quality of life assessment for acute heart failure patients from emergency department presentation through 30 days after discharge: A pilot study with the Kansas City Cardiomyopathy Questionnaire. J Card Fail. 2014;20(5):378 e11-5. . 25089314

[27] Cho EN, von Kanel R, Marten-Mittag B, Ronel J, Kolb C, Baumert J, et al Determinants and trajectory of phobic anxiety in patients living with an implantable cardioverter defibrillator. Heart. 2012;98(10):806–12. Epub 2012/05/01. 10.1136/heartjnl-2011-301204 . 22543838

[28] Kamphuis HC, de Leeuw JR, Derksen R, Hauer RN, Winnubst JA. Implantable cardioverter defibrillator recipients: quality of life in recipients with and without ICD shock delivery: a prospective study. Europace. 2003;5(4):381–9. . 14753636 10.1016/s1099-5129(03)00078-3

[29] Thomas SA, Friedmann E, Gottlieb SS, Liu F, Morton PG, Chapa DW, et al Changes in psychosocial distress in outpatients with heart failure with implantable cardioverter defibrillators. Heart Lung. 2009;38(2):109–20. 10.1016/j.hrtlng.2008.05.005 . 19254629

[30] Wassnig NK, Gunther M, Quick S, Pfluecke C, Rottstadt F, Szymkiewicz SJ, et al Experience With the Wearable Cardioverter-Defibrillator in Patients at High Risk for Sudden Cardiac Death. Circulation. 2016;134(9):635–43. 10.1161/CIRCULATIONAHA.115.019124 . 27458236 PMC4998124

[31] Amiaz R, Asher E, Rozen G, Czerniak E, Glikson M, Weiser M. Do implantable cardioverter defibrillators contribute to new depression or anxiety symptoms? A retrospective study. Int J Psychiatry Clin Pract. 2016;20(2):101–5. Epub 2016/04/08. 10.3109/13651501.2016.1161055 . 27052573

