# Supplementary material for: Anxiety, depression and quality of life in acute high risk cardiac disease patients eligible for wearable cardioverter defibrillator: Results from the prospective multicenter CRED-registry
Source: PLoS One. 2019 Mar 11;14(3):e0213261. doi: 10.1371/journal.pone.0213261 (PMC6411111; doi:10.1371/journal.pone.0213261)
Supplement: S2 Fig — (DOCX) [file pone.0213261.s002.docx]

**S2 Fig: Correlation of ∆MCS with ∆BDI-II score** **(A) and** **∆PCS and ∆BDI-II score** **(B)** (n=97).

(A)

(B)
