# Supplementary material for: Anxiety, depression and quality of life in acute high risk cardiac disease patients eligible for wearable cardioverter defibrillator: Results from the prospective multicenter CRED-registry
Source: PLoS One. 2019 Mar 11;14(3):e0213261. doi: 10.1371/journal.pone.0213261 (PMC6411111; doi:10.1371/journal.pone.0213261)
Supplement: S4 Fig — (DOCX) [file pone.0213261.s004.docx]

**S4 Fig: Baseline and 6-weeks depressive (A+B) and anxiety (C+D) symptoms by WCD therapy in patients without acute myocardial infarction, ICD explantation and ICD indication with postponed implantation** (N=71).

(A)

(B)

(C)

(D)
