# Supplementary material for: Anxiety, depression and quality of life in acute high risk cardiac disease patients eligible for wearable cardioverter defibrillator: Results from the prospective multicenter CRED-registry
Source: PLoS One. 2019 Mar 11;14(3):e0213261. doi: 10.1371/journal.pone.0213261 (PMC6411111; doi:10.1371/journal.pone.0213261)
Supplement: S1 Table — (DOCX) [file pone.0213261.s005.docx]

Supplementary Table 1: Baseline characteristics in patients with available baseline and follow-up questionnaire data and those alive without complete questionnaire data.

|  | Patients with questionnaire follow-up | Patients without questionnaire follow-up | p-value |
| --- | --- | --- | --- |
| n | 97 | 20 |  |
| Age [years ± SD] | 58 ± 14 | 62 ± 13 | 0.17 |
| Female gender [%] | 24 (n=23) | 40 (n=8) | 0.17 |
| Married [%] | 14 (n=14) | 10 (n=2) | 1.00 |
| At least certificate of secondary education [%] | 42 (n=41) | 35 (n=7) | 0.80 |
| Prior history of depression [%] | 6 (n=6) | 20 (n=4) | 0.07 |
| Current intake of psychotropic medication** [%] | 16 (n=16) | 15 (n=3) | 1.00 |
| Indication |  |  |  |
| Temporarily explanted ICD/ postponed ICD implantation [%] | 7 (n=7) | 10 (n=2) | 0.65 |
| Ischemic cardiomyopathy [%] | 26 (n=25) | 37 (n=7) | 0.41 |
| Non ischemic cardiomyopathy [%] | 67 (n=65) | 55 (n=11) | 0.32 |
| Comorbidity |  |  |  |
| Hypertension [%] | 48 (n=47) | 75 (n=15) | 0.05 |
| Hyperlipoproteinaemia [%] | 13 (n=13) | 15 (n=3) | 1.00 |
| Prior stroke [%] | 4 (n=4) | 5 (n=1) | 1.00 |
| Diabetes mellitus [%] | 24 (n=23) | 35 (n=7) | 0.40 |
| Prior myocardial infarction [%] | 22 (n=21) | 45 (n=9) | 0.05 |
| Current smoker [%] | 59 (n=57) | 75 (n=15) | 0.21 |
| Any regular alcohol intake [%] | 33 (n=32) | 30 (n=6) | 1.00 |
| Prior or active malignancy [%] | 7 (n=7) | 0 (n=0) | 0.60 |
| Arrhythmic risk profile  (syncope or family history of sudden cardiac death or prior resuscitation/ventricular tachycardia/ventricular fibrillation) [%] | 25 (n=24) | 40 (n=8) | 0.18 |
| clinical data |  |  |  |
| NYHA class [± SD] | 3.0 ± 0.7 | 3.1 ± 0.6 | 0.53 |
| Left-ventricular ejection fraction  [% ± SD] | 24.7 ± 6.9 | 27.5 ± 5.4 | 0.06 |
| Heart rate [1/min ± SD] | 79 ± 17 | 86 ± 13 | 0.06 |
| Systolic blood pressure [mmHg ± SD]  Dystolic blood pressure [mmHg ± SD] | 117 ± 19 | 114 ± 17 | 0.48 |
|  | 73 ± 13 | 70 ± 11 | 0.23 |
| Body mass index [kg/m² ± SD] | 27 ± 6 | 28 ± 4 | 0.43 |
| laboratoric parameters |  |  |  |
| Glomerular filtration rate  [ml/min/1.73m² ± SD | 68.2 ± 26.3 | 70.4 ± 26.6 | 0.76 |
| NT-pro-BNP [ng/l ± SD] | 7245 ± 12305 | 11870 ± 10088 | 0.27 |
| TSH [± SD] | 2.9 ± 7.1 | 2.9 ± 2.8 | 0.98 |
| Drug treatment |  |  |  |
| ACE-inhibitor or angiotensin-receptor blocker [%] | 92 (n=89) | 95 (n=19) | 1.00 |
| Beta-blocker [%] | 88 (n=85) | 60 (n=12) | < 0.01 |
| Mineralcorticoidreceptor antagonist [%] | 74 (n=72) | 40 (n=8) | < 0.01 |
| Thiazide or loop diuretics [%] | 81 (n=79) | 65 (n=13) | 0.13 |
| Baseline questionaires |  |  |  |
| BDI-II Score [± SD] | 9.3 ± 5.4 | 12.3 ± 7.0 | 0.08 |
| depressive symptoms [%] | 21 (n=20) | 25 (n=5) | 0.76 |
| State anxiety Score [± SD] | 39.9 ± 11.4 | 46.1 ± 11.7 | 0.04 |
| Trait anxiety Score [± SD] | 35.4 ± 9.8 | 41.4 ± 10.6 | 0.03 |
| Anxiety [%] | 52 (n=50) | 65 (n=13) | 0.32 |
| PCS [± SD] | 41.2 ± 11.2 | 38.2 ± 9.9 | 0.23 |
| MCS [± SD] | 49.4 ± 11.6 | 49.3 ± 11.9 | 0.96 |

ACE: angiotensin converting enzyme, ICD: implantable cardioverter defibrillator, NT-pro-BNP: N-terminal-pro-brain natriuretic peptide, NYHA: New York Heart Association, TSH: thyroid stimulating hormone, WCD: wearable cardioverter defibrillator

*data are presented as mean ± standard deviation (SD) or percentage [frequency n]

**selective serotonin reuptake inhibitors, benzodiazepines, low and high potency antipsychotics, tricyclic and other antidepressants
